# Patients and Parents’ Experience of Multi-Family Therapy for Anorexia Nervosa: A Pilot Study

**DOI:** 10.3389/fpsyg.2021.584565

**Published:** 2021-01-28

**Authors:** Victoria Baumas, Rafika Zebdi, Sabrina Julien-Sweerts, Benjamin Carrot, Nathalie Godart, Lisa Minier, Natalie Rigal

**Affiliations:** ^1^Psychiatric Unit, Institut Mutualiste Montsouris, Paris, France; ^2^Département de Psychologie, Université Paris-Nanterre, Nanterre, France; ^3^Université de Reims Champagne Ardenne, C2S EA 6291, Reims, France; ^4^CESP, INSERM, UMR 1018, University Paris-Sud, UVSQ, University Paris-Saclay, Villejuif, France; ^5^UFR des Sciences de la Santé Simone Veil (UVSQ), Versailles, France; ^6^Fondation de Santé des Etudiants de France, Paris, France

**Keywords:** multi-family therapy, anorexia nervosa, patients’ experience, parents’ experience, focus group

## Abstract

Background: Family therapy is considered as the gold standard in treatment of adolescents with anorexia nervosa (AN). Among the different types of family therapy, multi-family therapy (MFT) is increasingly used for treating AN, and shows promising results. In this article, our focus relied on the patients’ and their parents’ perceptions of the effectiveness and the underlying mechanisms of the MFT. Methods: The present pilot exploratory qualitative study included two focus groups conducted using a semi-structured approach: one with the adolescents (*n* = 3), and another with one or two of their parents (*n* = 4 mothers; *n* = 2 fathers). The subjects discussed were the changes observed in both AN symptoms and family interactions following therapy, and the mechanisms underlying these changes. We crossed the perspectives of the adolescents and of the parents on these two points. Results: Qualitative analysis revealed that while both adolescents and parents had difficulties relating the changes they observed in the last year to MFT, they were able to say that the group cohesion had several positive effects and that their family dynamics had improved. In the light of analysis the adolescents perceived more improvements related eating disorders symptoms than their parents did, while parents were concerned about a negative effect of MFT on their children. Discussion: While both patients and parents perceived improvements in both AN symptoms and family interactions in the past year, it was not clear if they considered MFT to have led to these improvements. FG also explored the MFT mechanisms underlying changes. Both adolescents and their parents stressed the beneficial effects of identification to others members of the group and shared experience to overcome social isolation. Parents also mentioned the sympathy they felt for each other. The idea that they give a central place to families in the therapy was also described by the families.

## Introduction

Anorexia nervosa (AN) is a psychiatric disorder affecting 0.3% of men, and 0.5 to 2.2% of women, with the highest prevalence among 15–18 year-old girls (Roux et al., 2013). AN is characterized by restriction of intake leading to significantly low body weight, persistent behavior to prevent weight gain, disturbed perception and experience of one’s own body, weight and shape, and the excessive fear of gaining weight or becoming fat (American Psychiatric Association et al., 2015). The multiplicity of AN’s consequences requires multidisciplinary care, amongst which family therapy is strongly recommended by practice guidelines, especially for children and adolescents (Haute Autorité de Santé, 2010).

Among the different forms of family therapy, multi-family therapy (MFT) is increasingly used in the treatment of eating disorders (ED). While literature is still scarce, results of Gelin et al. (2018)’s literature review regarding MFT efficacy in treating ED are promising: all nine studies (both prospective and retrospective) showed an improvement of ED symptoms, quality of life, self-esteem and mood for both patients and their parents. Moreover, the parents’ sense of efficacy and of parenting competence were enhanced. Those studies also highlighted a strong satisfaction of the families regarding MFT and a low rate of therapy drop out. At last, frequency and duration of patients’ hospitalizations were reduced than usual. Considering that around 10% of families do not adhere to single-family therapy, or even refuse this approach, MFT offers a valuable alternative (Asen, 2002; Cook-Darzens et al., 2005).

Multi-family therapy is a form of therapy that brings together several families (usually four to seven) affected by the same pathology. Initially used in the treatment of schizophrenia, MFT was initiated by Laqueur and his collaborators in the 1960’s (Le Grange and Eisler, 2009). MFT focuses its therapeutic effect on the whole family system and not only on the designated patient. The entire family is therefore involved in the treatment and becomes an active agent of change (Gelin et al., 2015). In the treatment of ED, MFT is inspired by different models including psychoeducational, systemic, psychodynamic, and attachment theories (Gelin et al., 2015). The main objective of MFT is to reduce AN symptomatology, in particular by directly involving parents in the management of their child’s eating behavior (Asen, 2002). MFT also aims to improve intra-family relationships and to reduce conflicts, particularly those related to eating (Le Grange and Eisler, 2009). The success of MFT mainly relies on the establishment of a group cohesion, which permits a feeling of understanding and mutual identification within the group. This process reduces families’ social isolation. Asen and Scholz (2010) described the following processes underlying MFT:

- Create solidarity. This is a source of support and it creates resources for families.
- Overcome stigma and social isolation. Families come out of isolation by sharing their experiences. Sharing allows them to identify with each other and reduces loneliness.
- Stimulate new perspectives by comparing themselves to other families through their similarities, but also their differences. Questioning a family on how they manage situations can, for example, induce a new perspective on their way of seeing things.
- Learn from each other, in particular through sharing experiences, mutual support and identification with other families.
- Mutual support and feedback promotes mutual learning.
- See the others as a mirror. Identifying with other families reduce isolation.
- Positive use of group pressure. Criticism and support from other group members promotes change.
- Discover and build on their skills, in particular by helping members of other families, but also by opening their feelings up to the group.
- Sharing and exchanging with several host families promotes learning by analogy and mutual support.
- Increase hope, in particular by bringing in families who have previously participated in a MFT and by planning for the future.
- Intensifying interactions and experiences makes it possible to understand some behaviors, but also to increase the possibility of comparing and identifying with others.
- Develop new behaviors in a safe space. The secure space of the MFT sometimes allows patients to experiment with new behaviors.
- Strengthen introspection through mutual learning, solidarity and the exercises suggested during MFT. These will facilitate the expression of participants’ emotions and will allow them to better observe and analyze them.

In England, the Maudsley Hospital team assessed the experiences of six families involved in a 4-day MFT group and identified the processes of change that takes place in MFT (Voriadaki et al., 2015). In addition to rating scales and filling a daily journal, they set up focus groups (FG). Adolescents and their parents took part in separate FG. Both adolescents’ and parents’ FG highlighted that sharing similar experiences and mutual identifications was a key aspect in fostering changes. The adolescents’ FG showed that the presence of others specifically helped generate new perspectives. The parents’ FG highlighted a reduction of their feelings of isolation and powerlessness following the MFT. Both adolescents’ and parents’ FG also showed that role-playing increased empathy, motivation and mobilization of family resources. Finally, participants identified the benefits of expressing emotions within their family. FG highlighted the presence of different processes identified by Asen and Scholz (2010) such as learning and mutual support, the creation of solidarity and the development of an external perspective of reflection. Offord et al. (2006) also conducted a qualitative study that explored AN patients’ experiences of their treatment’s. They interviewed seven young adults treated for AN using semi-structured interviews. The analysis of their speeches has allowed the emergence of four main themes: (i) “Removal from normality versus connecting with the outside world,” (ii) “Treated as another anorexic versus a unique individual in distress,” (iii) “Control and collaboration,” and (iv) “The importance of peer relationships.” The Interpretative Phenomenological Analysis revealed a strong sense of isolation during hospitalizations, a feeling of powerless and of being view simply as another anorexic. Some aspects of the treatment were therefore perceived as aggravating factors by the participants.

In this article are presented the results of two pilot focus-groups aiming to explore the experience of families focusing on the changes following a new 10-session MFT program in addition to the global therapeutic program, and the mechanisms underlying these changes according to these families. This ongoing study is an ancillary study of a multicenter randomized controlled trial comparing MFT to single-family therapy in adolescents with AN (Carrot et al., 2019).

## Materials and Methods

### Design

The present exploratory qualitative study included two focus groups. FG are a data collection procedure regrouping individuals around a predefined subject to focus on it (Kitzinger et al., 2004). Rarely used in clinical psychology, this procedure is yet an effective means of collecting participant’s personal opinions. FG usually brings together a dozen people for about an hour and a half around a pre-defined theme (Onwuegbuzie et al., 2009). Participants share common characteristics which allow them to form a homogeneous group. This homogeneity helps them sharing life experiences and feelings (Krueger and Casey, 2014). In addition, the interviewer’s non-directive conduct allows participants to develop and co-construct their ideas (Kalampalikis, 2004). FG can help completing quantitative data, which does not give access to in-depth and complete information and does not allow this co-construction of ideas. The communication space given by FG allows participants to build and structure an idea together and discuss around a theme.

### Participants

Participants were recruited from a larger RCT study (for a detailed protocol of the THERAFAMBEST study; see Carrot et al., 2019). In this study we used purposive sampling as we focused on one particular subgroup of the THERAFAMBEST study. Indeed, all participants attended the same MFT group. All four families who participated in the group were proposed to participate to the study. Two fathers refused to participate due to professional reasons. The parents of one adolescent did not give their consent for her to participate in the study. All three patients recruited (two girls and a boy) were aged 14 to 19 years and treated in outpatient care or hospitalized for AN in one of the four THERAFAMBEST study sites (Maison des Adolescents, Cochin Hospital, Paris, France). All four families were intact.

### Treatment

THERAFAMBEST’s MFT combines elements of the integrative model practiced by Cook-Darzens (2007) and elements of the Maudsley approach (Eisler, 2005). Four main themes are addressed in the program: (i) understanding and managing AN, (ii) family relations and family identity, (iii) overcoming social isolation, (iv) values, beliefs, and perceptions.

While some of the objectives are naturally achieved by the structure and dynamics of the group, other objectives are encouraged by specific interventions and therapeutic strategies and techniques. Tools used in sessions are based on several concepts and practices: cognitive behavioral approaches (psychoeducation, learning by analogy), medical family therapy (adaptation to processes of illness), psychodynamics (differentiation process through identification with other families), and systemic group approach and family therapy.

Multi-family therapy comprises 10 sessions over a year. Each session lasts three hours and involves initially five to seven families including the patients, their parents and non-systematically their siblings (but sometimes some family drop). Siblings attend to all sessions but three focus on parenting competences to cope with ED symptoms. The staff includes two lead co-therapists and several assistants (their number will depend on the size of the group). Assistants help families during the activities, providing further explanations and/or support if needed.

### Procedure and Materials

This study was approved by the French National Agency for Health and Medical Drugs (ANSM) and by the local Independent Ethics Committee (“Comité de Protection des Personnes” – Ile de France III; CPP: 2016-A00818-43). Information about the present study and the goals of the FG was verbally explained at the last session of the MFT group. The recruitment of FG is still undergoing as the saturation point is not yet reached. The data gathered were confidential and identifying information have been anonymized.

#### Focus Groups

Multi-family therapy is a group experience so we choose to use FG to explore interaction and group dimension. In order to cross the perspectives of parents and adolescents, two FG discussions were conducted: one with the adolescents (FG-A) and one with their parents (FG-P). This choice was made in order to encourage the participants’ free expression. Furthermore, a previous study showed that parents and adolescents may have different opinions on MFT (Voriadaki et al., 2015). The FG were carried out the same day one month after the end of the last MFT session by four women. RZ and NR are university lecturers in charge of the present ancillary study. LM is a researcher (PhD in psychology) in charge of the THERAFAMBEST participants’ evaluations, and therefore met with the participants beforehand. VB is a master student in psychology doing an internship on the THERAFAMBEST study. During the last MFT session, NR was the one proposing to the families to participate to the focus group. Both focus groups took place in the Institut Mutualiste Montsouris’ psychiatric unit and not in the patients’ usual hospital.

The aim of the FG was to identify the various changes perceived as a result of the treatment, but also the factors that lead to these changes according to the families. During the FG, the moderators asked questions in line with the interview guide presented in Table 1. The interview guide was informed by a rigorous review of the literature on AN, which identified themes and sub-themes related to changes and factor of changes. The highlighting of these themes and sub themes were expected during the FG. Each discussion lasted approximately an hour and a half. In order to analyze their content, they were audio-recorded and transcribed with the participants’ consent but it was not sent back to the participants to check or make corrections of the content. Thanks to the recording, an exact transcription of the participants’ discourse was made, which also justifies the absence of note-taking during the FG.

**TABLE 1:** Grid interview.

**Changes**	
*AN symptoms*	- Did you observe any change in the AN symptoms following the MFT? - Following the MFT, did you observe any change in your child’s concerns about his/her weight/your concerns about your weight?* - Following the MFT, did you observe any change in your child’s concerns about food/your concerns about food?* - Following the MFT, did you observe any change in your child’s concerns about his/her body shape/your concerns about your body shape?* - Following the MFT, did you notice any change in your child’s food rituals (such as sorting or cutting food in small pieces, or taking with small bites for examples)/your food rituals (such as sorting or cutting food in small pieces, or taking with small bites for examples)?* - Following the MFT, did you notice any change in your child’s hyperactivity/your hyperactivity?*
*Family interactions*	- Did you observe any change in your family relationships following the MFT? - Following the MFT, did you notice any change in the quality of the interactions within your family?* - Following the MFT, did your family do more fun activities?* - Following the MFT, did emotions were more shared within your family?* - In your opinion, has the MFT changed your family’s vision of the future?* - Following the MFT, did you observe any change in the way you behave with your child during meals/in the way your parents behave with you during meals?* - In your opinion, did MFT promote or improve your feelings of parental competence?* - Following the MFT, did you notice any change in your children relationship/in your relation with your brothers and/or sisters?*
**Agents of change**	
*Group cohesion*	- In your opinion, what allowed the changes we just discussed? - In your opinion, does the group dimension of the MFT could have facilitated changes we previously discussed?* - In your opinion, did the MFT allow mutual understanding and empathy within the group?*
*Therapeutic alliance*	- Will you consider that you had a trustful relationship with the therapists? - In your opinion, did the relation with the therapists allowed any changes we discussed?

*^∗^These additional questions were asked if the adolescents or their parents did not discuss these topics by themselves.*

### Data Analysis

The two FG’s audiotapes were anonymously transcribed, and a thematic interpretation, which is an inductive analytic approach, was chosen to explore their content. This analysis strategy was designed to understand the complex system of meanings attached to a unique and subjective phenomenon. A standardized procedure guarantees the methodology rigor (Vaismoradi et al., 2013). First, each interview was read and the main themes were double-blind coded by two pairs of authors (RZ and LM; SJS and NR). Then, most frequent themes were identified and discussed by the four authors. In this study, we respected the criteria of scientific rigor established by the qualitative analysis (Fossey et al., 2002).

## Results

### Participants

Demographic and clinical characteristics of the patients included in the FG-A are presented in Table 2. FG-A included 2 girls and 1 boy aged 14 to 19 years old. While duration of the disease was similar between the three adolescents (2 to 3 years), age of first symptoms differed (12 to 16 years). When the MFT began, the two females were hospitalized and one male was treated in outpatient care. After the MFT, they all were outpatients. All patients’ BMI increased during MFT and only one of the girls has been hospitalized twice. The FG-P included six parents: four mothers, including one of an adolescent who did participate to the MFT but not to the FG, and two fathers.

**TABLE 2:** Demographic and clinical characteristics of the adolescents included in the FG-A.

	**Adolescent 1 (A1)**	**Adolescent 2 (A2)**	**Adolescent 3 (A3)**
Gender	Female	Female	Male
Age	16	14	19
Duration of AN (years)	2	2	3
Number of past hospitalizations (total duration in weeks)	3 (35)	3 (13)	5 (57)
Care before MFT	Inpatient	Inpatient	Outpatient
Care after MFT	Outpatient	Outpatient	Outpatient
Number of hospitalizations during MFT (total duration in weeks)	0	2 (40)	0
Body Mass Index (BMI) before MFT	19.23	14.81	19.84
BMI after MFT	20.83	16.43	20.28

### Major Themes Found in Both of the FG-A and -P

#### Difficulty Linking Patients’ Evolutions and MFT

Both parents and adolescents could not establish a clear link between the changes they observed during the past year and the MFT.

Mother 1: “MFT is one of many factors that have contributed to an evolution, but we are unable to say in which proportion it contributed. I think it has not been useless but I will be unable to tell you how useful it has been for me.”

#### Being in a Group Has Several Positive Effects

Both parents and adolescents argued that the group was a source of support and hope, as they could exchange with people living similar difficulties. Discussions within the group highlighted resources of each participant. It also broke isolation and reduced the parents’ feeling of guilt.

Father 1: “Overall, we come out of it more positive; I found the group effect to encourage us to be more optimistic. That is pretty good. So, it gives a bit of hope. Each time, after a session of this family therapy, I was more positive than when I went there.”
Adolescent 2: “Seeing other people who were in the same situation as I was, it helped me. It helped me to talk with them.”

The presence of the siblings was more controversial between the two groups. Adolescents perceived it as beneficial for their siblings while parents though that could have censored their children’s participation in the discussions.

Adolescent 3 (talking about his sibling): “I mainly noticed that during therapy’s sessions, they spoke to me more freely, and they identified themselves a lot with others. It was cool. Not necessarily in terms of how they behaved afterwards, but during the sessions they were involved”.
Mother 2 (talking about sibling’s support groups): “As they are amongst sibling, they can complain about their sick sister or brother. Because, it is not easy, they do not dare to do it in front of us, in front of other parents. Even less in front of their brother or sister. Well, I am saying that but I do not know what they told you.”

It should be noted that all participants mentioned the fact that they knew each other prior the MFT (patients met during previous hospitalizations and parents in a parents’ group) was probably facilitating the group cohesion.

#### MFT Improved the Family Dynamics

Participants noted an improvement in communication within the family as they understand each other better. They also mentioned a better understanding of the disease by the parents.

Mother 3: “I also felt very strongly that I was very touched by what the other adolescents could say. As a result, it helped me detach myself and I felt things that might have annoyed me if it came from my child, I would not have understood. I understood better through the words of another young person.”
Adolescent 1: “I guess that when I was sick they used to be extremely careful talking to me, now they say things freely.”
Adolescent 1: “I understood that it must be hell to have a child who does not eat anymore.”

#### Criticism of the Disparity in the Stages of the Disease Among the Patients

Parents and adolescents argued that the heterogeneity of the group in terms of stages of the disease was a limitation for two mean reasons: (i) it involves different needs and priorities for the families and (ii) it is challenging for the patients as it might leads to comparison.

Mother 1: “We are not at the same stage, thus we do not all have the same priorities”
Adolescent 1: “At one point, when I was finally getting out of it, and there was a therapy session, and there were other people who said they wanted to stop eating carbohydrate, I felt bad. […] Finally, I got out of it, and I think that it made it stronger, because there were comments like that. But it hurt me a little bit at the time to see people who were not at the same stage as I was.”

#### Frustration That Family Issues Could Not Be Discussed in the Group

Parents and adolescents expressed frustration regarding their families’ specific issues that could not be address within the group in a satisfying depth. They wished they could address more personal issues in a uni-familial therapy.

Father 1: “The family has never been put in the center, and it is normal. We are not going to expose all our problems in the group. But, there is a point where we have to address these issues.”
Adolescent 1: “Sometimes it was a bit frustrating because we could start to speak about an issue that we wanted to talk through, or we really wanted to get into more family centered issues. But as we were in a group and we were not there to talk about ourselves, well, we cut it short.”

### Major Themes Specific to the FG-A

#### Improvements Over the Last Year

Adolescents mentioned the improvements they observed over the past year in terms of ED symptoms: decreased preoccupation about weight, decreased guilt about eating, decreased compulsive eating, decreased perfectionism, and openness to other concerns than ED-related ones (notably seduction and romantic relationships). At last, concerning hyperactivity, adolescents noted a variable evolution: sometimes it improves, and sometimes there is a resurgence of symptoms. They also reported being more self-confident and an improvement in their mood.

Adolescent 1: “Now I am able to focus on something other than weight, sport, and food.”
Adolescent 1: “A year ago I was shy. In the eyes of others I had to be perfect, I controlled everything I said, I could not cross any line, now I do not care. I can party, I can dance, I can let loose with my friends, there is no more problem. Even with people I do not know.”
Adolescent 2: “I feel much better, I am less sad, I cry less, I see life in a less negative way. In the last year, there have been many changes.”

#### Changes in the Family Emotional Tone

Adolescents specifically mentioned changes in emotional expression and tone within their families: they reported more affection and humor.

Adolescent 1: “With my sister we laugh all the time, we have regained the complicity we had before, that is nice.”
Adolescent 3: “We are more affectionate. But not only in the words, well, in the words too. We have never been the type of people to say things to each other.”
Improvement of their parents’ well-being

Adolescents observed improvement in their parents’ well-being during the last year.

Adolescent 1: “My mother, she has not really changed her behavior except that she is more relaxed, she is not scared all the time anymore. […] My father is also more relaxed. He thinks about other things than me. They are no longer just the parents of a sick child.”

### Major Themes Specific to the FG-P

#### Parents’ Concerns About the Short-Term Deleterious Effect of MFT on Their Children

Parents expressed concerns about a possible short-term deleterious effect of the topics discussed in MFT on both their children suffering from AN and the siblings.

Mother 2: “However, I know that my daughter sometimes came out of the session worse than when she came in. In fact, it could have been a bit of a drag. Us parents, we were happy to come, but for them it was harder.”
Mother 2: “It was violent for the little ones, at least for the younger ones. I wish my youngest daughter had not been there.”
Difficulties coming to therapy encountered by the parents.

Parents mentioned the burden of the investment required for the medical care of their children and their difficulties in reconciling work and attending MFT.

Mother 1: “I was thinking: “We are going to spend another half day in MFT”. We have busy jobs. You cannot imagine how many half-days we had to free to go to medical appointments and therapies’ sessions here and there. It is hard to manage, we still have a job to do, and we have to get paid at the end of the month”.

#### Difficulties to Cope With the Topics Discussed in Therapy

Parents talked about their own difficulties in coping with the topics discussed in therapy, in parallel to those their children might have encountered.

Mother 3: “We are fine. In fact, I think we are holding up. But, maybe they had fewer defenses to cope with what they heard in there. And even for us, eh, it was upsetting.”

#### Comparison of MFT With Single-Family Therapy

Parents compared MFT to single-family therapy. According to them, MFT would allow a better adherence of ones being reluctant to single-family therapy. While single-family therapy would allow for more cohesion within the family. Parents concluded that these two kinds of family therapies are complementary.

Mother 4: “I think that if there had been single-family therapy, I think that it would not have worked, it would have been too hard to be face-to-face.”
Mother 3: “For the single-family therapy we had to drag the children, they did not want to go. But, each time, it created something strong and we were quite united for two or three hours. It did not do that after MFT. Each time we would say “well, let’s do something afterwards,” but each time everyone went about their business. MFT has not the same effects afterwards.”
Mother 3: “I think it takes both, in fact. We need spaces like this one where we actually highlight the resources we have. The disease has an effect on the family and our family and other families are the same; families are actually hurt by the disease. And then, sometimes, we also need other spaces where we can talk about what is difficult in a particular family. Because it might not be the same thing in our family and in another one, because I do not think that there is a typical anorexic child’s family.”

## Discussion

The objective of this research was to explore the experience of families following a 10-session MFT included a global treatment program for AN. Specifically, changes observed by families in both AN symptoms and family interactions following therapy, and the mechanisms underlying these changes were discussed in FG, were screened. Underlying mechanisms did not appear in the discussion because participants failed to rely improvements they observed and MFT therapeutic processes, except group cohesion. On the other hand, changes in symptomatology and in family dynamics were discussed. Finally, MFT-related issues appeared that was not anticipated.

Improvements in AN symptomatology did not appeared in the parents’ speech, but they were pointed out by the adolescents. Patients mentioned a decrease of their food rituals and preoccupations about body shape, weight and/or meals following MFT. Decrease of body-related concerns following MFT has been described in literature (Gelin et al., 2015). Results concerning problematic physical activity are mixed, as this symptom seems to fluctuate. However, adolescents did not seem to be able to explain this variation in relation to the therapy.

Both adolescents and their parents perceived improvements in their family relationships. Adolescents stressed the increase of closeness, humor, frankness and affection between each other, while parents focused on the increase of communication within the family. Furthermore, both parents and adolescents reported an improvement of their interactions during meal, which can be a challenging moment for families with AN adolescent. This is in accordance with Scholz et al. (2005)’s results that showed a decrease in conflicts and tension within families following MFT. Furthermore, this result is also important because the MFT in the THERAFAMBEST project was conceived with the aim, among others, of offering an eating-focused care to assist parents in their mission to feed their child (Carrot et al., 2019). Finally, families reported an increase of hope for the future following the MFT. This result thus confirms the idea that the increase in hope is promoted by the intervention of families in MFT (Asen and Scholz, 2010).

The positive effects provided by the group emerged from both focus groups. As it has been stressed by Voriadaki et al. (2015), the group cohesion is a key agent of change in MFT. Our results are in line with Asen and Scholz (2010)’s assumption that group cohesion is made possible by mutual identification, feelings of understanding and empathy (Asen and Scholz, 2010). Both adolescents of the present study and their parents stressed the beneficial effects of identification to others members of the group and shared experience to overcome social isolation. Parents also mentioned the sympathy they felt for each other. Parents of this MFT group were indeed very close: they frequently communicated and seen each other outside of the MFT group. During the FG, they shared their desire to stay in touch in the future. However, all participants mentioned some frustration associated to the fact that MFT is not focusing on one particular family’s issues. To give a central place to the notion of family in the therapy has been stressed as primordial in MFT (Cook-Darzens, 2007). It might be useful to orient them to single-family therapy in order to address issues that cannot be addressed in MFT. The two kinds of family therapy (single- versus multi-) could prove to have complementary effects. The results of Eisler et al. (2016)’s study indeed showed that the addition of MFT and single-family therapy allows for faster improvements (in terms of weight, presence of menstruation and absence of bulimic symptoms) than single-family therapy alone.

Various issues that were not anticipated emerged from the interviews. On the positive side, the improvement in the parents’ psychological state was described by the adolescents. This is an interesting finding because we know that adolescents can feel guilty about making family members suffer because of their illness, which is very pervasive (Voriadaki et al., 2015). On the negative side, parents insisted on the difficulty of reconciling the families’ agendas to get to the therapy sessions. They also mentioned their own difficulties to cope with content of certain sessions and their concerns about its effect on their children (both patients and siblings). Finally, adolescents and parents felt that the heterogeneity in the progress of the disease reinforced comparisons between patients rather than strengthened the group cohesion.

This pilot study completes the evaluation of THERAFAMBEST study (Carrot et al., 2019) by collecting the participants’ subjective experiences of MFT. This qualitative evaluation allow identifying both the benefits and the factors of improvement related to MFT perceived by the families. The qualitative method is complementary to the quantitative analysis as it allows assessing participants’ feelings and experiences (Aubin-Auger et al., 2008). Thus, qualitative methods promote a better understanding of lived experience or social phenomena. The different types of qualitative methodology (phenomenological, case study or grounded theory, etc.) involve data analysis and are intended to be just as rigorous as the quantitative method. A similar assessment will be carried out for single-family therapy to allow a comparison of the families’ experiences of these two forms of therapy.

This pilot study has several limitations. First, as it is an ongoing study, the size of the sample is limited. Furthermore, the fact that two of the three adolescents showed significant improvements before the beginning of the MFT might not be representative of the families attending to MFT. The recruitment of FG will continue until the saturation point will be reached.

## Conclusion

While this group was not representative of the AN population as a whole, these FG gave some indications about the experience of families in MFT. The group cohesion appears to be a key agent of change in MFT according to the families. The therapy seems to have consolidated adolescents’ progress that occurred before its beginning. Analysis of a more diverse sample of families might highlight different kind of benefits allowed by MFT in accordance with the family’s needs.

## Data Availability Statement

The raw data supporting the conclusions of this article will be made available by the authors, without undue reservation.

## Ethics Statement

The studies involving human participants were reviewed and approved by this study was approved by the French National Agency for Health and Medical Drugs (ANSM) and by the local Independent Ethics Committee (“Comité de Protection des Personnes” – Ile de France III; CPP: 2016-A00818-43). Written informed consent to participate in this study was provided by the participants’ legal guardian/next of kin.

## Author Contributions

NR designed the ancillary focus group study in collaboration with VB, RZ, SJ-S, and LM. BC is the principal investigator of the THERAFAMBEST study. NG is the scientific director of the THERAFAMBEST study and co-investigator. LM is a co-investigator of the THERAFAMBEST study. All authors read and approved the final manuscript. VB and RZ contributed equally as first authors and LM and NR contributed equally at the latest authors. All authors contributed to the article and approved the submitted version.

## Conflict of Interest

The handling editor declared a shared affiliation with several of the authors VB, RZ, SJ-S, and NR at the time of review. The remaining authors declare that the research was conducted in the absence of any commercial or financial relationships that could be construed as a potential conflict of interest.
